# Placental Lipases in Pregnancies Complicated by Gestational Diabetes Mellitus (GDM)

**DOI:** 10.1371/journal.pone.0104826

**Published:** 2014-08-12

**Authors:** Helen L. Barrett, Marta H. Kubala, Katherin Scholz Romero, Kerina J. Denny, Trent M. Woodruff, H. David McIntyre, Leonie K. Callaway, Marloes Dekker Nitert

**Affiliations:** 1 UQ Centre for Clinical Research, University of Queensland, Herston, QLD, Australia; 2 Royal Brisbane and Women's Hospital, Herston, QLD, Australia; 3 School of Medicine, University of Queensland, Herston, QLD, Australia; 4 Mater Research Institute, University of Queensland, Brisbane, QLD, Australia; 5 School of Biomedical Sciences, University of Queensland, St Lucia, QLD, Australia; University of Missouri, United States of America

## Abstract

Infants of women with gestational diabetes mellitus (GDM) are more likely to be born large for gestational age with a higher percentage body fat. Elevated maternal lipids may contribute to this. Placental lipases such as lipoprotein lipase (LPL), endothelial lipase (EL) and hormone sensitive lipase (HSL) are involved in transferring lipids from mother to fetus. Previous studies of expression of these lipases in placentae in women with diabetes in pregnancy have reported divergent results. Intracellular lipases such as adipose triglyceride lipase (ATGL), and HSL are central to lipid droplet metabolism. The activities of these lipases are both influenced by Perilipin 1, and ATGL is also activated by a co-factor comparative gene identification-58 (CGI-58) and inhibited by G_0_/G_1_ switch gene 2 (GS02). None of these modifying factors or ATGL have been examined previously in placenta. The purpose of this study was therefore to examine the expression of ATGL, HSL, LPL, EL, as well as Perilipin 1, GS02 and CGI-58 in term pregnancies complicated by GDM. mRNA and protein expression of the lipases were measured in placentae from 17 women with GDM and 17 normoglycaemic pregnancies, matched for maternal BMI and gestational age of delivery. ATGL mRNA expression was increased and HSL mRNA expression reduced in placentae from GDM although there was no differences in protein expression of any of the lipases. All lipases were localised to trophoblasts and endothelial cells. The expression of Perilipin 1 and CGI-58 mRNA was increased and GS02 not altered in GDM. These results suggest that there is no difference in expression in these four lipases between GDM and normoglycaemic placentae, and therefore altered lipid transfer via these lipases does not contribute to large for gestational age in infants of women with GDM.

## Introduction

In pregnancy, the mother supplies multiple fuel sources for infant growth, including glucose, free fatty acids, ketone bodies and amino acids [1]. Both maternal glucose and maternal lipids have been associated with infant birth weight and rates of large for gestational age infants [2]. [3], [4]. Gestational diabetes mellitus (GDM) is associated with increased infant birth weight and adiposity [5], [6]. Infants born LGA to women with GDM have a higher percent body fat than those born LGA to women with uncomplicated pregnancies [7].

Maternal metabolism in the third trimester is altered to ensure an adequate supply of nutrients to the fetus. These alterations include increased maternal lipoprotein concentrations and an increase in the triglyceride content of maternal lipoproteins [1]. The components of maternal lipoproteins are taken up into the placenta via the activity of lipoprotein receptors, lipases, fatty acid binding proteins and other mechanisms allowing supply of triglycerides and cholesterol to the fetus [8], [9]. Aspects of cholesterol transport to the fetus has been shown to be altered in the setting of GDM [10] and expression of phospholipid transfer protein mRNA increased [11].

The human placenta has been shown to express lipoprotein lipase (LPL), endothelial lipase (EL) and hormone-sensitive lipase (HSL). Other triglyceride gene family members, including hepatic lipase, pancreatic lipase, pancreatic lipase-related protein 1 and 2 have not been detected [12]. There is discordance between the reported expression of LPL, EL and HSL in several maternal disease states, possibly due to differences in metabolic control [13], high maternal TG or FFA [14], [15] or in maternal obesity [16].

Adipose triglyceride lipase (ATGL) expression has not been examined in placenta. ATGL is central to the lipolysis of triacylglycerols in intracellular lipid droplets [17]. It undertakes the initial and rate limiting step in hydrolysing triacylglycerols to diacylglycerols and free fatty acids (FFA). ATGL mediated active lipolysis is facilitated by an activating co-factor Comparative Gene Identification-58 (CGI-58) [18] and inhibited by G_0_/G_1_ switch gene 2 (GS02) [19]. Perilipin 1 (Perilipin A), a lipid droplet associated protein, regulates both ATGL mediated and HSL mediated lipolysis. Perilipin 1 influences ATGL mediated lipolysis by sequestering CGI-58, releasing it upon phosphorylation during active lipolysis and HSL mediated lipolysis. This most likely occurs by a binding interaction between HSL and perilipin 1 assisting the access of HSL to lipids in the lipid droplet [20]. An alteration in ATGL in placenta could impact upon the transport of triglycerides to the fetus and hence fetal growth and adiposity.

The current study examines the placental expression of ATGL and its activators and inhibitors, HSL, EL and LPL in placentae from women with GDM and uncomplicated pregnancy.

## Methods

### Subjects

Pregnant women were recruited from a tertiary general and obstetric hospital and gave written informed consent. Permission for the study was granted by Royal Brisbane Human Research Ethics Committee and Women's Hospital and The University of Queensland, Human Research Ethics Committee. Participants gave written informed consent. Participants were matched for maternal BMI, gestational age of delivery and adjusted birth weight centile. Maternal BMI was calculated from an early pregnancy weight (routinely obtained at the first booking in visit, typically 10–16 weeks gestation) divided by the squared height in meters. The customized birth centile was calculated with the online calculator gestation.net (www.gestation.net). Diagnosis of gestational diabetes mellitus was defined by current Australasian Diabetes in Pregnancy Society guidelines [21]. Placental tissue pieces were collected immediately post-delivery, sampled randomly (∼1 cm^3^), with sampling performed away from areas of infarction or calcification. The samples were kept at −80°C until analysis. In addition, 1 cm^3^ samples of placenta for paraffin embedding were washed in PBS, placed into 4% paraformaldehyde for 48 hours and kept in 70% ethanol solution until embedding for immunohistochemistry.

### RNA isolation and quantitative real-time PCR

mRNA was isolated from placenta with the Allprep RNA/DNA extraction mini kit (Qiagen, Chadstone, VIC, Australia). Tissue was first disrupted by violent shaking with a 5 mm stainless steel bead in a TissueLyser (Qiagen, Chadstone, VIC, Australia). RNA was quantified by Nanodrop and all samples had 260/280 ratios >1.8. 750 ng mRNA was reverse transcribed to cDNA with the QuantiTect reverse transcription kit (Qiagen, Chadstone, VIC, Australia) using a mixture of oligodT and random primers. Quantitative real-time PCR was performed on 18.75 ng of cDNA with 300 nM of primers and iTaq universal SYBR green mastermix (Bio-Rad, Gladesville, NSW, Australia) on an iQ5 PCR machine (BioRad). The PCR protocol consisted of 1 cycle at 95°C for 10 min, 40 cycles of 95°C for 15 sec and 59°C for 1 min followed by dissociation curve analysis. Primers unique for the target gene and covering exon-exon junctions were designed with primerBLAST. The primer sequences are presented in Table S1 (see supplementary file). Gene expression was normalized to the housekeeping gene β-Actin (*ACTB*). To adjust for potential differences in cellular composition of the placental samples, gene expression was also normalized to the geometric mean of expression of the housekeeping gene TATA-box binding protein (*TBP*), cytokeratin 7 (*CK7*) as a marker for trophoblast cells, CD34 (*CD34*) for endothelial cells and desmin (*DES*) for smooth muscle cells.

### Protein expression

Placenta were lysed with a RIPA buffer consisting of 50 mM Tris, 1% Triton-X, 0.1% SDS, 0.5% DOC, 150 mM NaCl, and protease inhibitor cocktail (Roche, Applied Science, VIC, Australia). Tissue was disrupted by violent shaking with a 5 mm stainless steel bead in a TissueLyser (Qiagen, Chadstone, VIC, Australia). After lysis, the sample was centrifruged for 10 min at 4°C and the protein content in the supernatant was determined by bicinchoninic acid assay (Sigma-Aldrich, Castle Hill, NSW, Australia). 30 µg of protein was loaded onto a 4–12% gradient NuPAGE Bis-Tris gel (Life Technologies, Mulgrave, VIC, Australia), transferred onto a polyvinylidene difluoride (PVDF) membrane (Millipore, Kilsythe, VIC, Australia) and blocked for 1 hour with 5% non-fat dry milk in PBS-Tween. Primary antibody for rabbit anti-LPL (1∶300, sc-32885 Santa Cruz Biotech, Texas, USA), rabbit anti-HSL (1∶150, sc-25843 Santa Cruz Biotech, Texas, USA), rabbit anti-EL (1∶150, 100030 Cayman chemical, Michigan, USA), or rabbit anti-ATGL (1∶300, 2138 Cell Signalling Technology, Massachusetts, USA were co-incubated with mouse anti-β-Actin (1∶20000, A5316, Sigma Aldrich, Castle Hill, NSW Australia) overnight at 4°C with agitation. Secondary LI-COR antibodies, goat anti-rabbit 800CW (1∶10000, 926-32211, LI-COR) and donkey anti-mouse 680LT (1∶15000, 926-68022, LI-COR) were incubated for 1 hour at room temperature and protein was detected by the Odyssey Infrared Imaging System (LI-COR). Lipase protein expression was analyzed by densitometry correcting for differences in protein loading by using β-actin levels.

### Immunohistochemistry

Paraffin-embedded sections (5 µm) were baked, and rehydrated. Antigen retrieval was performed by heating to 125 degrees °C in 100 mM sodium citrate, 0.05% Tween 20 at pH 6.0 for 30 minutes. Endogenous peroxidase activity was blocked with hydrogen peroxide 3% for 10 mins followed by 15 mins with Biocare Background Sniper (MACH2, Biocare Medical, Concord CA). Immunolabelling was performed using polyclonal rabbit antibodies to LPL, anti-HSL, anti-EL antibodies (Biorbyt, Cambridge, UK: LPL (1∶1000, orb13546), EL (1∶500, orb100394), HSL (1∶100, orb40070)) and ATGL antibody (1∶100, Cell Signalling Technology, Massachusetts, USA #2138). Confirmatory immunohistochemistry for LPL was performed with a second polyclonal rabbit antibody (1∶1000, Santa Cruz Biotech, sc-32885). After washing, the slides were incubated with a biotinylated polyvalent goat secondary antibody followed by DAB incubation for 1 minute. Slides were counterstained with Harris' Haematoxylin (HHS 16, Sigma Aldrich) and mounted with coverslips.

### Image analysis

HSL protein expression was analysed with a quantitative immunohistochemistry method described by Helps et al [22]. This method uses Ruifrok and Johnston's color deconvolution image processing method to digitally separate hematoxylin and DAB staining. The imaging processing and analysis was performed in NIH-ImageJ software using Landini's ImageJ plugin then histogram analysis and a weighting calculation to estimate the amount of DAB staining. We took 10 randomly selected frames from each of 4 control and 4 GDM placentae that were processed and stained concurrently. Within each frame, the placental villi were demarcated by hand on the NIH-ImageJ software.

### Statistical analysis

Experiments were performed in duplicate. Data are presented as mean +/− SEM unless stated otherwise. Differences between groups were examined with two tailed Mann-Whitney U tests (Prism version 5.03 software (GraphPad, La Jolla, CA)). Significance was set at <0.05. Correlation analysis was performed with Spearman's rho testing.

## Results

### Study participants

Placentae from 17 women with GDM and 17 normoglycemic (control) women with uncomplicated pregnancy were collected. The women were matched for maternal BMI, gestational age at delivery, adjusted birth weight centile and infant sex. Maternal and pregnancy characteristics are shown in Table 1. Other than the 1 hour glucose following a 50 g glucose challenge, there were no significant differences between the groups. Mean maternal HbA1c in the women with GDM measured toward the end of the third trimester was 5.3% (SEM 0.17). The majority of women with GDM (n = 12) were diet controlled, with 4 needing insulin therapy and one prescribed metformin. Sensitivity analyses were performed excluding and including the women on GDM needing pharmacotherapy, which did not alter the results.

**Table 1:** Maternal and pregnancy characteristics.

	Control	GDM	P
n	17	17	-
Maternal age (years)	32.6 (4.6)	32.7 (5.1)	1.0
Maternal BMI in early pregnancy mean(SD)	28.4 (6.8)	31.3 (9.8)	0.57
Caucasian ethnicity n (%)	16 (94)	14 (82)	-
1Hr glucose following 50 g GCT mean(SD)	5.75 (1.27)	8.74 (1.45)	<0.001
Gestational age of delivery (weeks)	38.7 (0.8)	39.3 (1.0)	0.05
Birth weight (g)	3468 (387.0)	3619 (364.5)	0.36
Birth weight centile*	56.5 (30.0)	54.5(26.55)	0.80
Infant sex (F/M)	8/9	10/7	-

* Adjusted birthweight centile [38].

### ATGL

**Figure 1:**
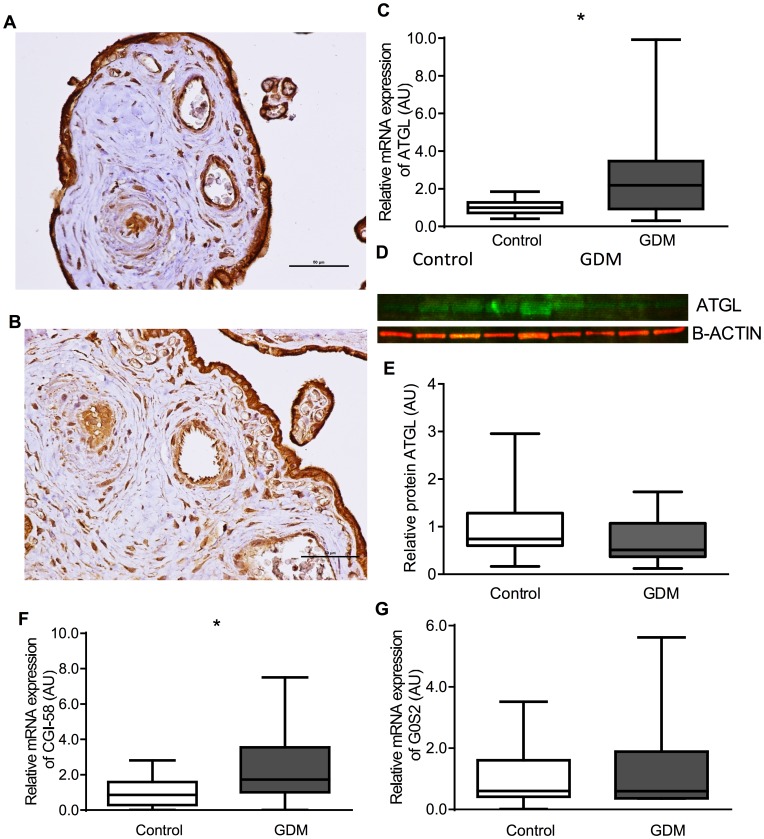
ATGL. Showing immunohistochemistry (A Control, B GDM), C relative mRNA expression of ATGL, D representative western blot images of ATGL, E relative protein expression of ATGL, F relative mRNA expression of CGI-58, G relative mRNA expression of GS02.

The relative expression of ATGL mRNA was increased (GDM median 2.19 AU (IQR 0.93–3.48) vs control (1.00 AU (0.72–1.27), P = 0.02). ATGL was localized to syncytiotrophoblasts and endothelial cells but also to stromal cells including Hofbauer cells and decidual cells (Figure 1A,B). There was no clear difference in the protein expression between placentae from control women or those with GDM for ATGL (Figure 1C,D). The relative mRNA expression of CGI-58 was increased (GDM median 1.33 AU (IQR 1.05–3.54) vs control (0.87 AU (0.30–1.60), P = 0.03) and that of GS02 unchanged (GDM median 0.93AU (IQR 0.36–2.21) vs control (0.60 AU (0.41–1.60), P = 0.35).

### HSL

**Figure 2:**
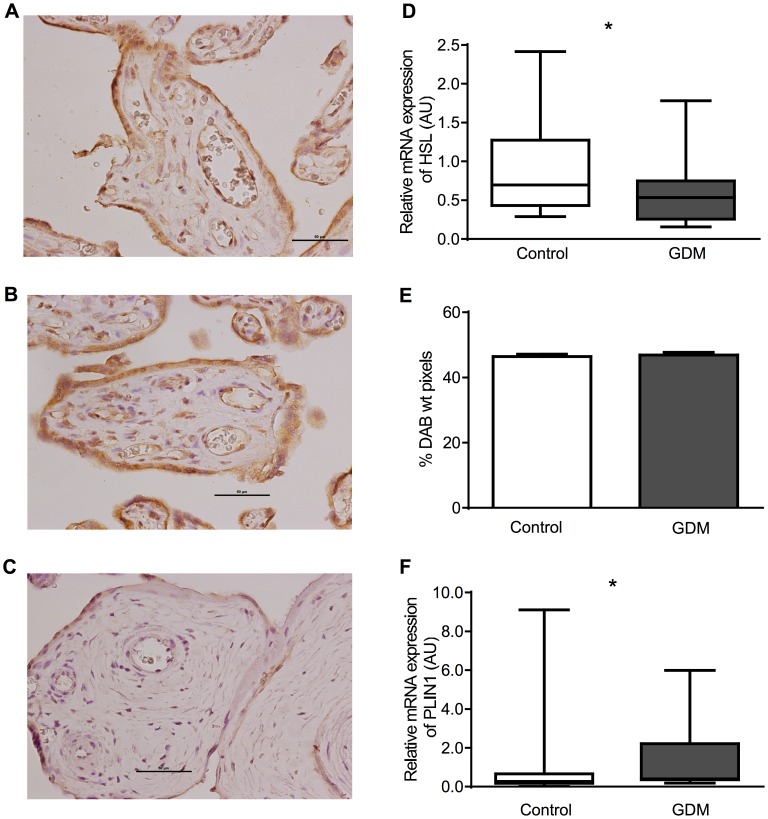
HSL. Showing immunohistochemistry (A Control, B GDM, C negative control), D relative mRNA expression, E relative protein expression as measured by quantitative immunohistochemistry.

The relative expression HSL mRNA was decreased (GDM median 0.53 AU (0.26–0.74) vs control 0.70 AU (0.43–1.27), P = 0.05) and that of Perilipin 1 increased (GDM median 0.40 AU (IQR 0.36–2.22) vs control 0.28 AU (0.18–0.67), P = 0.02) in placentae from women with GDM. Protein expression for HSL was localized to syncytiotrophoblasts and endothelial cells and also to stromal cells including Hofbauer cells and decidual cells (HSL: Figure 2A,B, Control: Figure 2C). HSL protein expression was not quantifiable by western blot with either antibody. HSL expression was analysed by quantitative immunohistochemistry (2E) and there was no difference found.

### LPL and EL

**Figure 3:**
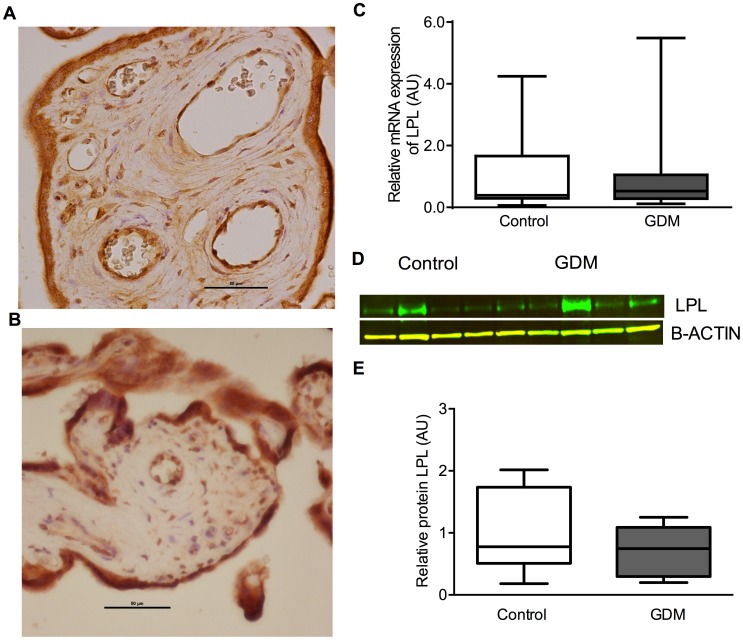
LPL. Showing immunohistochemistry (A Control, B GDM), C relative mRNA expression, D representative western blot images, E relative protein expression.

**Figure 4:**
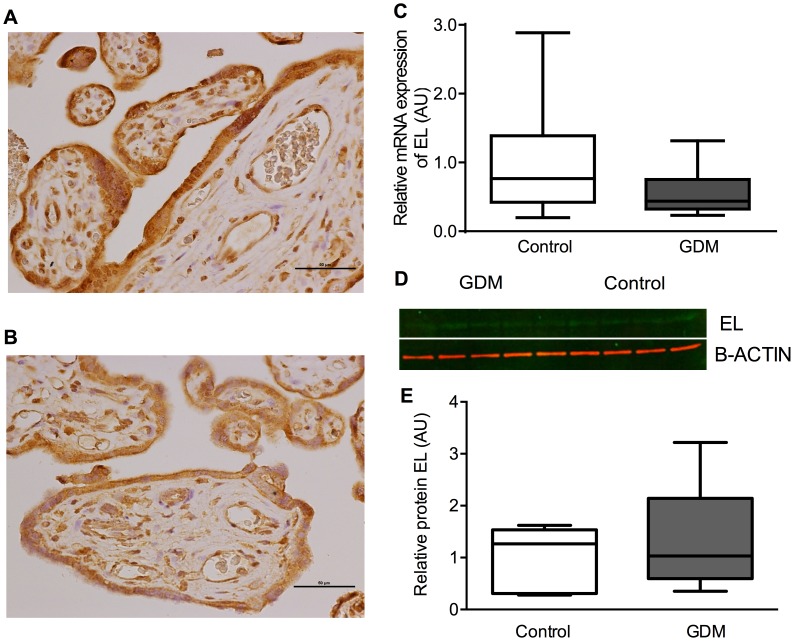
EL. Showing immunohistochemistry (A Control, B GDM), C relative mRNA expression, D representative western blot images, E relative protein expression.

There was no difference in mRNA expression between GDM and control placentae for LPL (GDM median 0.53 (IQR 0.28–1.06) vs control 0.39 AU (0.30–1.67), P = 0.83) and EL (GDM 0.44 AU (IQR 0.32–0.75) vs control 0.77 AU (0.42–1.39), P = 0.11). Protein expression for LPL and EL was localized to syncytiotrophoblasts and endothelial cells and also to stromal cells including Hofbauer cells and decidual cells (LPL (Figure 3A,B) and EL (Figure 4A,B)).There was no clear difference in the protein expression between placentae from control women or those with GDM for either LPL (Figure 3C,D) or EL (Figure 4C,D).

### Relationship with clinical factors

There was no significant correlation between mRNA expression for any of the lipases, CGI-58, GS02 or perilipin 1 and early pregnancy maternal BMI or birth weight (data not shown). HSL mRNA expression was significantly associated with adjusted birth weight centile (Spearman rho 0.34 (95%CI −0.02–0.63), P = 0.05)). None of the other variables were significantly associated with adjusted birth weight centile. In women with GDM, LPL mRNA expression was negatively correlated with HbA1c (Spearman's rho −0.79 (95%CI −0.93–−0.42), P = 0.001). No other lipase mRNA expression was significantly associated with HbA1c.

## Discussion

The current study, examining placentae from women with well-controlled GDM and BMI matched women with uncomplicated pregnancies, has demonstrated for the first time the presence of ATGL mRNA and protein in human placenta. All lipases examined localised to the maternal and fetal sides of the placenta as well as the Hofbauer cells and decidual cells. ATGL mRNA was increased and HSL mRNA decreased in placentae from women with GDM compared to controls, but no difference in protein expression or localisation between GDM and control placentae was detected for any of the four lipases described in this study. The present study also shows that mRNA for the lipolytic regulatory molecules CGI-58 and Perilipin 1 were upregulated in GDM placenta whereas there was no change in expression of G0S2 mRNA.

ATGL undertakes the first step in lipolysis, hydrolysing triacylglycerol to diacylglycerol. CGI-58 is an activating co-factor, increasing the activity of ATGL [18] and GS02 an inhibitor [19]. In patients with type 2 diabetes mellitus on insulin treatment, ATGL protein was borderline increased in subcutaneous adipose tissue, and G0S2 mRNA and protein were decreased when insulin treatment was withheld and patients were hyperglycaemic [23]. ATGL mRNA expression in omental adipose tissue has been found to be reduced in insulin resistant obesity [24] but not altered in subcutaneous or omental adipose tissue in insulin sensitive obesity [25]. Increased CGI-58 expression in placentae from women with GDM could indicate an increased activation of lipolysis. ATGL mRNA expression was also increased although no difference in protein levels was detected.

The decrease in HSL mRNA found in the current study is not in keeping with the previously reported increased HSL mRNA expression in placentae from women with type 1 diabetes and suboptimal metabolic control [13]. We found no difference in HSL protein expression as measured by quantitative immunohistochemisty. Women with GDM in our study achieved an HbA1c (5.3%) similar to the 5.4% HbA1c of the control women in the Lindegaard study. The study by Lindegaard [13] found that HSL mRNA expression increased with increasing HbA1c, whereas we found no association. Insulin down regulates HSL activity [26]. In women with type 1 diabetes, poor metabolic control, as defined by an increasing HbA1c would relate to a degree of insulin deficiency. GDM, rather than being a state of insulin deficiency as in type 1 diabetes is a state of insulin resistance [27]. We do not have insulin levels for the women in our study but it could be suggested that the decrease of HSL mRNA found in the current study could be explained by the excellent glycaemic control achieved and a presumed degree of insulin resistance.

ATGL and HSL are both intracellular lipases. Unspecified intracellular lipase activity has been described in placenta. Intracellular lipase activity has been examined in type 1 diabetes (11 women), type 2 diabetes (4 women) and impaired glucose tolerance (IGT) 7 women) [28]. In IGT/type 2 women an intracellular lipase with optimal activity at pH 4 (possibly lysosomal acid lipase) was increased significantly compared with controls and there was a correlation between this placental lipase activity and birth weight in IGT/type 2 women. [28]. Another lipase, with optimal pH at 6.0, has been demonstrated in placental cytosol and in the microvillous membrane [29]. The activity of this lipase did not differ between control placentae and those from women with type 1 diabetes mellitus, but was decreased in smokers. The presence of both ATGL and HSL in the placenta indicates that as in other tissues, rates of lipolysis are likely to be regulated through the amount and activity of both lipases.

Perilipin 1, which the current study found to be present in the placenta, is important in lipid droplet metabolism. In basal states, perilipin coats the lipid droplet which limits access of lipases to the droplet. In stimulated states (fasting/exercise/β -adrenergic stimulation), perilipin 1 is phosphorylated and facilitates HSL related lipolysis [20]. Perilipin also influences ATGL activation as it is bound to CGI-58 and releases CGI-58 when phosphorylated. CGI-58 can then interact with ATGL. In patients with insulin requiring type 2 diabetes mellitus, perilipin 1 mRNA and protein levels were reduced when the insulin was withheld and patients were hyperglycemic compared to when they were on insulin and euglycemic [23]. In obesity, Perlilipin 1 mRNA and protein expression have been variably reported to be the same, higher or reduced and it likely varies with gender, degree of obesity and adipose tissue depot [30], [31]. At term, LPL mRNA, protein and activity have been detected in villous tissue [10], [32], [33], isolated trophoblasts [34], and homogenised placental samples [13], [35]. The current study found no difference in LPL mRNA or protein between placentae from women with GDM compared to control. Previous studies have shown varying results, possibly related to the degree of maternal glycemic control [13], [36] and maternal obesity [10], [33], [34]. The women in the current study all had good glycaemic control and maternal BMI was matched between the two groups, possibly explaining the lack of difference seen here in LPL expression. In agreement with the findings of the current study, most [13], [35], [37] but not all [12] immunohistochemisty studies localise LPL in term placenta to syncytiotrophoblasts, endothelial cells and macrophage like Hofbauser cells.

Endothelial lipase mRNA is increased in placentae from women with type 1 diabetes mellitus [13] and obese women with GDM compared to lean women with GDM or normoglycemic pregnancy [16]. In the current study, we found no difference in mRNA or protein expression between placentae from women with GDM and normoglycaemic women. This may be due to the good glycemic control of the women with GDM and matched BMI between the groups. In keeping with the current study, EL has been consistently localised to endothelial cells and syncytiotrophoblasts in human placenta [13], [16], [35].

Some limitations of the current study should be noted. While the matched maternal BMI of the two groups in this study removes the potential confounding effect of maternal BMI, it also limits our ability to explore the potential different contribution of GDM and maternal obesity. However, we do have a wide range of maternal BMI in both groups and despite this, could not demonstrate any correlation with maternal BMI and lipase mRNA expression. The current study does not include assessment of lipase activity. It is possible that lipase activity could differ where mRNA and protein expression do not.

The current study has demonstrated the presence of ATGL mRNA, and protein in human term placenta, and its localization to both maternal and fetal sides of the placenta. It has also shown the presence of some of the important regulators of lipid droplet lipolysis. There was no difference seen in any lipase expression between placentae from BMI matched women with well controlled GDM and normoglycemic pregnancy. This lack of difference suggests that in well controlled GDM, these lipases do not underlie the increased infant adiposity seen.

## Supporting Information

Table S1 **Primer Sequences for qPCR assays.**
(DOCX)
