# Supplementary material for: Placental Lipases in Pregnancies Complicated by Gestational Diabetes Mellitus (GDM)
Source: PLoS One. 2014 Aug 12;9(8):e104826. doi: 10.1371/journal.pone.0104826 (PMC4130608; doi:10.1371/journal.pone.0104826)
Supplement: Table S1 — Primer Sequences for qPCR assays. (DOCX) [file pone.0104826.s001.docx]

Supplementary Table 1 Primer sequences

| Gene name | Forward primer | Reverse primer |
| --- | --- | --- |
| *LPL* | 5’-TGGATCGCTCCACTTTGACC | 5’-GGGCTTCGGACTGGTAAACA |
| *LPIG* | 5’-GTCCAGCCCCTGCTATCTCA | 5’-CCTTTTCAAACTGACCCTTGCC |
| *LIPE* | 5’-CACATTAGACCCAGAAGATGCC | 5’-GGCAGCGAAACTTGACAGTG |
| *PNPLA2* | 5’-TGCCCACTTTGTGTGTATGTG | 5’-CCAGGAGTGCGACGCT |
| *PLIN1* | 5’-GAAGTTGAAGCTTGAGGAGCG | 5’-GGCTTCCTTAGTGCTTGGTGT |
| *ABHD5* | 5’-GAACGACCAGACCTTGCTGA | 5’-GCTGTCTCACCACTTGGAGT |
| *G0S2* | 5’-CACTAAGGTCATTCCCGCCT | 5’-AGCACGTACAGCTTCACCAT |
| *CK7* | 5’- CCGTGCGCTCTGCCTATGGGG | 5’- GCTCCAGAAACCGCACCTTGTCGAT |
| *CD34* | 5’- CCACAGGAGAAAGGCTGGGCGA | 5’- AGCCCCTCGGTTCACACTGGC |
| *DES* | 5’- TCCGAGAAACCAGCCCTGAGCAA | 5’- GTGGCCTCACTGACGACCTCCC |
| *ACTB* | 5’-AGCGAGCATCCCCAAAGTT | 5’-GGGCACGAAGGCTCATCATT |
